# A Strategy to Enhance Humidity Robustness of p‐Type CuO Sensors for Breath Acetone Quantification

**DOI:** 10.1002/smsc.202200096

**Published:** 2023-02-28

**Authors:** Dina N. Oosthuizen, Ines C. Weber

**Affiliations:** ^1^ Particle Technology Laboratory Department of Mechanical & Process Engineering ETH Zurich CH-8092 Zurich Switzerland; ^2^ Department of Endocrinology, Diabetes, and Clinical Nutrition University Hospital Zurich CH-8091 Zurich Switzerland

**Keywords:** breath analyses, humidity robustness, low-power devices, nanotechnology, p-type sensors

## Abstract

Low‐cost metal oxide sensors are highly attractive for emerging applications such as breath analysis. Particularly promising are p‐type sensors that can operate at low temperatures, a key requirement for compact and low‐power devices. To date, however, these sensors lack sufficient sensitivity, selectivity, and humidity robustness to fulfil stringent requirements faced in real applications. Herein, a flame‐made and low‐power sensor (operated at 150 °C) that consists of CeO_2_‐decorated CuO nanoparticles is introduced, as determined by X‐ray diffraction and X‐ray photoelectron spectroscopy analysis. Most remarkably, this sensor features excellent robustness to 10–90% relative humidity. This is attributed to the presence of CeO_2_ nanoclusters, which may act by scavenging OH^−^ and allow the readsorption of oxygen onto the CuO surface. To demonstrate its immediate impact, this sensor is investigated for the detection of acetone, a biomarker for fat burning. It detects acetone with high sensitivity (i.e., 50 ppb) and features excellent acetone selectivity (>9.8) toward key inorganic interferants (i.e., NH_3_, H_2_, and CO). Most importantly, the CeO_2_–CuO sensor accurately quantifies acetone concentrations in the exhaled breath of 16 volunteers (bias and precision of 90 and 457 ppb). As a result, it is attractive for low‐power and humidity robust detection of volatiles in breath analysis.

## Graphical abstract

A p‐type low‐temperature CuO sensor quantifies acetone in the exhaled breath of 16 volunteers in excellent agreement with mass spectrometry. It exhibits high robustness to varying relative humidity and highly concentrated inorganic interferants due to the addition of CeO_2_ nanoclusters on CuO sensors. Hence, this sensor is promising for low‐power fat‐burning monitoring.

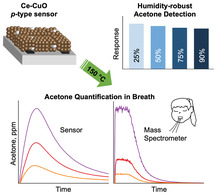

## 1 Introduction

Semiconducting metal oxide (SMO_*x*_) sensors are promising for emerging applications including air^[^ ^1^ ^]^ and food quality monitoring,^[^ ^2^ ^]^ search and rescue,^[^ ^3^ ^]^ as well as breath analysis.^[^ ^4^ ^]^ They can be fabricated in a low‐cost, highly sensitive, and compact manner,^[^ ^5^ ^]^ making them ideal candidates for portable devices in continuous monitoring. Yet, despite myriad of sensing materials available, SMO_*x*_ sensors are hardly found in real applications. This is due to their high operation temperatures (typically above 300 °C), hindering their use as low‐power (e.g., battery driven) devices, in combination with high requirements regarding sensitivity, selectivity, and humidity robustness.

A model compound to demonstrate this is breath acetone. Acetone is a biomarker for fat burning^[^ ^6^ ^]^ to track metabolic changes (e.g., during exercise^[^ ^7^ ^]^ or dieting^[^ ^8^ ^]^) and assist in the treatment of metabolic diseases (e.g., diabetes,^[^ ^9^ ^]^ obesity,^[^ ^6^ ^]^ or epilepsy^[^ ^10^ ^]^). Its detection, however, is hindered by strict requirements posed by breath analysis, specifically the presence of high relative humidity (RH, can reach >89% in breath^[^ ^11^ ^]^), high sensitivity (acetone in breath can be as low as 148 parts per billion [ppb]^[^ ^12^ ^]^), and high selectivity (exhaled breath contains more than 1000 compounds^[^ ^13^ ^]^). While recent works demonstrate breath acetone detection with SMO_*x*_ sensors (e.g., Si/WO_3_^[^ ^14^ ^]^), these sensors are typically operated at high temperatures (e.g., >350 °C), hindering their integration into low‐power devices. Hence, reducing sensor operation temperatures while at the same time meeting sensitivity and selectivity requirements is a key bottleneck of today's sensors.

Optimizing material–analyte interactions at the nanoscale has enabled a variety of n‐type and p‐type sensing materials that detect acetone already below 200 °C (**Table** **1**). These, however, do not fulfil the stringent requirements of breath analysis for multiple reasons: first, humidity effects are often not considered (upper part of Table 1), despite their crucial importance in breath analysis. Second, they often lack sufficient sensitivity to detect sub‐parts‐per‐million (ppm) acetone concentrations. Third, most of these sensors feature limited selectivity toward inorganic interferants or did not report selectivity at all. Hence, it is not surprising that low‐temperature acetone sensors have rarely been tested on real breath. In fact, only two low‐temperature sensors were tested on breath (i.e., Pt/Sb_2_O_3_–Fe_2_O_3_^[^ ^15^ ^]^ and BaSnO_3_,^[^ ^16^ ^]^ Table 1), but as their cross‐sensitivity to humidity is missing, as well as validation with state‐of‐the‐art instruments (e.g., gas chromatography–mass spectrometry (GC–MS)), these results are inconclusive.

**Table 1:** Comparison of acetone sensors operating at low temperatures (≤200 °C) with a lowest limit of quantification (LOQ) below 10 ppm

Material (dopant conc. in mol%)		Temp. [°C]	RH [%]^a)^	LOQ [ppm]^b)^	^c)^Selectivity			Breath	References
					NH_3_	H_2_	CO		
Without humidity									
n‐type	ZnO	RT	–	2.0	–	–	–	–	[67]
	ZnO	RT	–	1.0			–	–	[68]
	Ce‐ZnO	RT	–	1.0	2.8 (0)^c)^	–	–	–	[69]
	V_2_O_5_	RT	–	1.0	1.5 (0)	–	–	–	[70]
	BaSnO_3_	80	–	5.0	–	–	–	Yes^d)^	[16]
	WO_3_–SnO_2_	170	–	0.1	–	–	–	–	[71]
	(0.5) Rh–SnO_2_	200	–	1.0	–	–	–	–	[72]
	Fe_2_O_3_/Al–Zn	200	–	1.0	–	38(0)	–	–	[73]
	Gd–Fe_2_O_3_	200	–	1.0	16 (0)	–	–	–	[74]
	Zn–Fe_2_O_4_	200	–	10.0	–	–	8.0 (0)	–	[75]
	WO_3_/Pt–GNs	200	–	10.0	–	–	–	–	[76]
p‐type	Ti_3_C_2_T_*x*_	RT	–	1.0	–	–	–	–	[77]
	Pt/Sb_2_O_3_–Fe_2_O_3_	RT	–	0.9	–	–	–	Yes^d)^	[15]
	PbS	RT	–	9.3	–	–	12.5 (0)	–	[78]
	Co_3_O_4_	160	–	1.0	–	–	–	–	[79]
	CuO	170	–	10.0	–	–	–	–	[80]
	PrFeO_3_	180	–	10.0	21 (0)	–	–	–	[81]
	ZnCo_2_O_4_	200	–	0.5	18 (0)	18 (0)	18 (0)	–	[82]
	(1.25) Pd–LaFeO_3_	200	–	0.8	–	–	–	–	[83]
With humidity									
n‐type	ZnO/NiO	RT	56	1.0	–	–	–	–	[84]
	ZnO/SnO_2_	110	90	0.5 (0)					[85]
	Pt–In_2_O_3_	180	85	0.3 (0)	–	–	–		[32]
	(1) Pd‐loaded SnO_2_	200	50	1.0	–	–	–	–	[86]
p‐type	K_2_W_7_O_22_	RT	30	0.3	–	–	–	–	[33]
	Amorphous Sb_2_S	RT	40	10.0	–	–	–	–	[87]
	MXene/rGO/CuO aerogels	RT	60	10.0	–	–	–	–	[88]
	MoO_3_	RT	90	0.5	–	–	–	–	[89]
	La_1‐*x*_Sr_*x*_CoO_3_	RT	68	10.0 (45)	–	–	–	–	[90]
	Co_3_O_4_	RT	89	1.0 (0)	–	–	–	–	[91]
	(1) Au‐loaded LaFeO_3_	100	70	2.5 (0)	–	–	12 (0)	–	[92]
	Pt–Fe_2_O_3_	139	75	0.2 (0)	–	–	–	–	[93]
	Co_3_O_4_	150	80	1.0 (90)	–	–	–	–	[94]
	p‐TiO_2_	150	75	0.5	–	–	–	–	[95]
	(0.5) Ru–NiO_2_	200	90	5.0	–	–	–	–	[96]
	(0.1) Pt–Co_3_O_4_	200	80	0.5	–	–	21.5 (0)	–	[97]
	(5) CeO_2_–CuO	150	90	0.05	30	100	9.6	Yes	^e)^

a) Highest humidity reported.

b) LOQ is the smallest amount of an analyte measured at given RH, if not, RH specified in brackets.

c) Selectivity ((*R*_acetone_−1)/(*R*_interferant_−1)) calculated from response at given RH, if not, RH specified in brackets.

d) No quantification or validation given.

e) This work.

It is worth noting that a majority of the low‐temperature acetone sensors tested in the presence of humidity feature p‐type behavior (lower part of Table 1). In fact, p‐type SMO_*x*_ sensors are known for their ability to perform at low temperatures,^[^ ^17^ ^]^ even room temperature,^[^ ^18^ ^]^ due to the narrow indirect bandgap (e.g., from 1.2^[^ ^19^ ^]^ to 1.4 eV^[^ ^20^ ^]^ for CuO) and the majority charge carrier type (i.e., holes, h^+^ due to metal vacancies^[^ ^19^ ^]^). In addition to this, literature reveals that metal oxide doping (e.g., with CeO_2_ on In_2_O_3_^[^ ^21^ ^]^ or SnO_2_^[^ ^22^ ^]^) can further reduce humidity cross‐sensitivity. Hence, building on p‐type sensors and using metal oxide doping is a promising strategy to address the above humidity and temperature limitations and improve low‐power sensors to enable measurements in real environments.

Here, a relatively low‐temperature (i.e., 150 °C) sensor is presented that consists of flame‐made copper oxide containing cerium oxide (CeO_2_–CuO). Copper oxide was chosen due to its p‐type sensing behavior at low temperatures^[^ ^23^ ^]^ and potential for acetone detection at low concentrations (e.g., 320 ppb^[^ ^24^ ^]^), while CeO_2_ addition is known to improve humidity robustness.^[^ ^21^ ^]^ Sensors were produced with flame spray pyrolysis (FSP), as this allows: 1) formation of highly porous sensing films with enhanced sensitivity,^[^ ^25^ ^]^ 2) close control of particle characteristics (e.g., size, morphology, crystal phase), as well as 3) offering flexibility in material composition at the nanoscale.^[^ ^26^ ^]^ The sensor performance was evaluated at various RHs (i.e., 0–90%) for acetone concentrations ranging from 50 to 2000 ppb. In addition, sensor selectivity toward highly concentrated (and thus problematic) inorganic breath interferants (e.g., H_2_, CO, and NH_3_) as well as their mixtures was assessed, next to ethanol, isoprene, methane, and H_2_S. To better understand the underlying sensing performance, material characterization by X‐ray diffraction (XRD), transmission electron microscopy (TEM), nitrogen (N_2_) adsorption, as well as X‐ray photoelectron spectroscopy (XPS) was carried out. Finally, as a proof of concept, these sensors were tested on the exhaled breath of 16 volunteers, and breath acetone was validated with proton‐transfer‐reaction time‐of‐flight mass spectrometry (PTR‐ToF‐MS), following previous protocols.^[^ ^27^ ^]^

## 2 Results and Discussion

### 2.1 Gas Sensing

**Figure 1:**
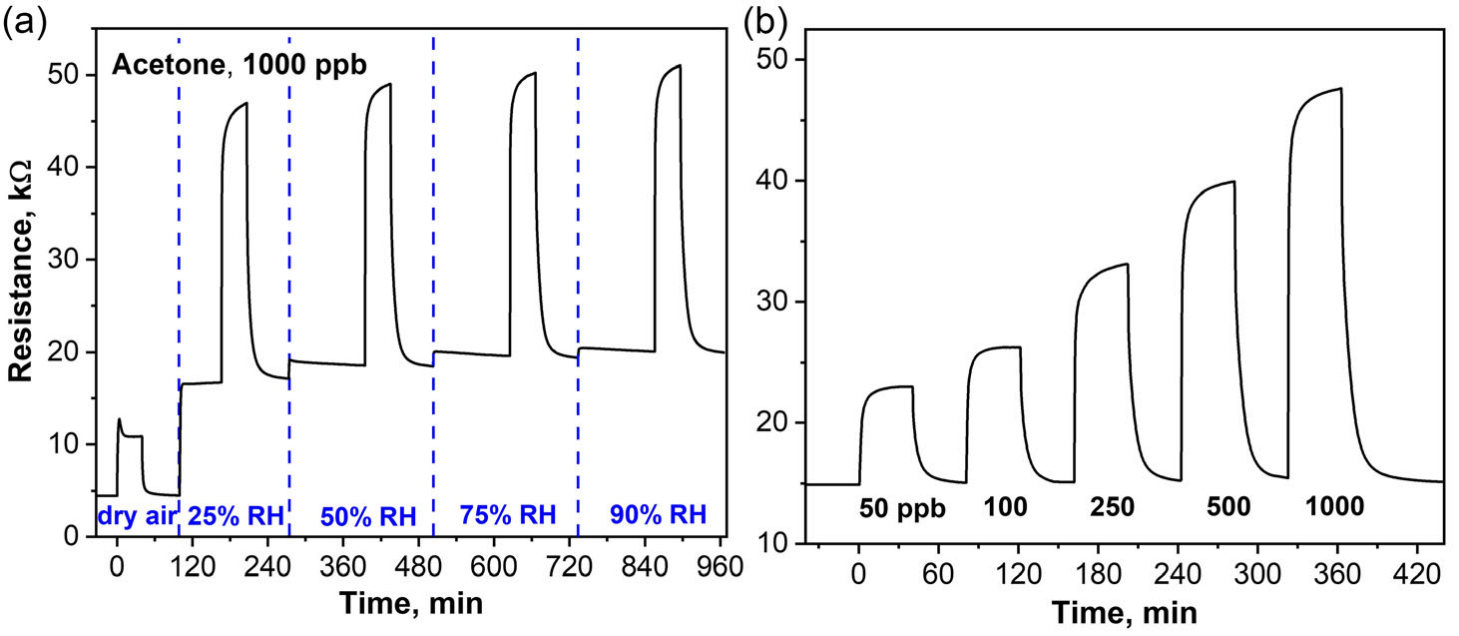
a) CeO_2_–CuO film resistance upon exposure to 1000 ppb acetone in dry air, 25, 50, 75, and 90% RH conditions. b) Exposure to breath‐relevant 50–1000 ppb acetone in 90% RH. Note that the sensor was operated at 150 °C.

First, the humidity cross‐sensitivity (i.e., humidity robustness) of the CeO_2_–CuO sensor was investigated. This is an important parameter due to the varying humidity content in the environment and in breath (>89% RH^[^ ^11^ ^]^). **Figure** **1**a shows the sensor resistance when exposed to 1000 ppb acetone in dry air and at various RHs (i.e., 25, 50, 75, and 90%). The sensor exhibits typical p‐type conductivity, in which the sensor resistance increases upon exposure to acetone (reducing gas) and recovers to the initial baseline resistance in air (oxidizing atmosphere).^[^ ^28^ ^]^ Most importantly, the effect of different humidities is nearly negligible for the sensor, demonstrated by the small variation (<10%) in sensor response (S = *R*_g_/*R*_a_, *R*_g_: resistance in gas, *R*_a_: baseline resistance in air) and sensor resistance *R*_a_ between 25 and 90% RH. However, note that the *R*_a_ in dry air varied significantly (i.e., <30% from 90% RH), likely due to the initial decrease of ionosorbed oxygen in the presence of humidity.^[^ ^29^ ^]^ This is, however, unproblematic in real‐life measurements, as we show below in the section on breath analysis (Section 2.3).

In fact, when testing an identically prepared pure CuO thin film (without any CeO_2_), it was largely affected by the presence of humidity (i.e., 30% deviation in sensor response between dry air and 90% RH, Figure S1, Supporting Information). This was also observed for other FSP‐prepared CuO thin films (i.e., *R*_a_ changed by 25% from dry air to 50% RH^[^ ^30^ ^]^) and thick films (i.e., *R*_a_ changed by 12% from dry air to 50% RH^[^ ^29^ ^]^). Therefore, the low humidity cross‐sensitivity of the CeO_2_–CuO sensor is attributed to the presence of CeO_2_ clusters, as observed previously with CeO_2_–In_2_O_3_^[^ ^21^ ^]^ and CeO_2_–SnO_2_ sensors.^[^ ^22^ ^]^ This possibly comes from the capacity of CeO_2_ to act as OH^−^ scavenger and allows oxygen readsorption on the CuO surface as in the case of In_2_O_3_^[^ ^21^ ^]^ and SnO_2_.^[^ ^22^ ^]^

Next, the CeO_2_–CuO sensor was exposed to acetone concentrations between 50 and 1000 ppb in 90% RH (Figure 1b) at 150 °C, as this covers a typical range of basal acetone concentrations in healthy people that can go as low as 148 ppb.^[^ ^12^ ^]^ Most importantly, even the lowest concentrations (i.e., 50 ppb) were clearly distinguishable with a high signal‐to‐noise ratio (i.e., SNR > 1000 for three identically prepared sensors). The sensor exhibited a nonlinear response dependence (power law function) to the acetone concentration (Figure S2a, Supporting Information), which is in close agreement with diffusion reaction theory for semiconducting sensors.^[^ ^31^ ^]^ The sensitivity (i.e., 50 ppb) is superior to other low‐temperature sensors, where the lowest measured concentration was 300 ppb (i.e., with a Pt–In_2_O_3_ and K_2_W_7_O_22_ sensor in dry air^[^ ^32^ ^]^ and 30% RH,^[^ ^33^ ^]^ respectively), which is insufficient to resolve the smallest acetone concentrations in breath.^[^ ^12^ ^]^ In addition, the CeO_2_–CuO sensor showed good repeatability (i.e., sensor response deviation of 2.2%) to four consecutive pulses of 1000 ppb acetone at 90% RH (Figure S2b, Supporting Information). The response (*τ*_res_) and recovery (*τ*_rec_) times for 1000 ppb acetone were 340 and 670 s, respectively. Note that the sensor response remained rather stable for 21 days (i.e., average response of 2.69 ± 0.16, Figure S3, Supporting Information), while it decreased at longer times (i.e., 1.65 after 200 days and 1.26 after 320 days). To account for such differences, the sensor may be calibrated regularly before measurements.

**Figure 2:**
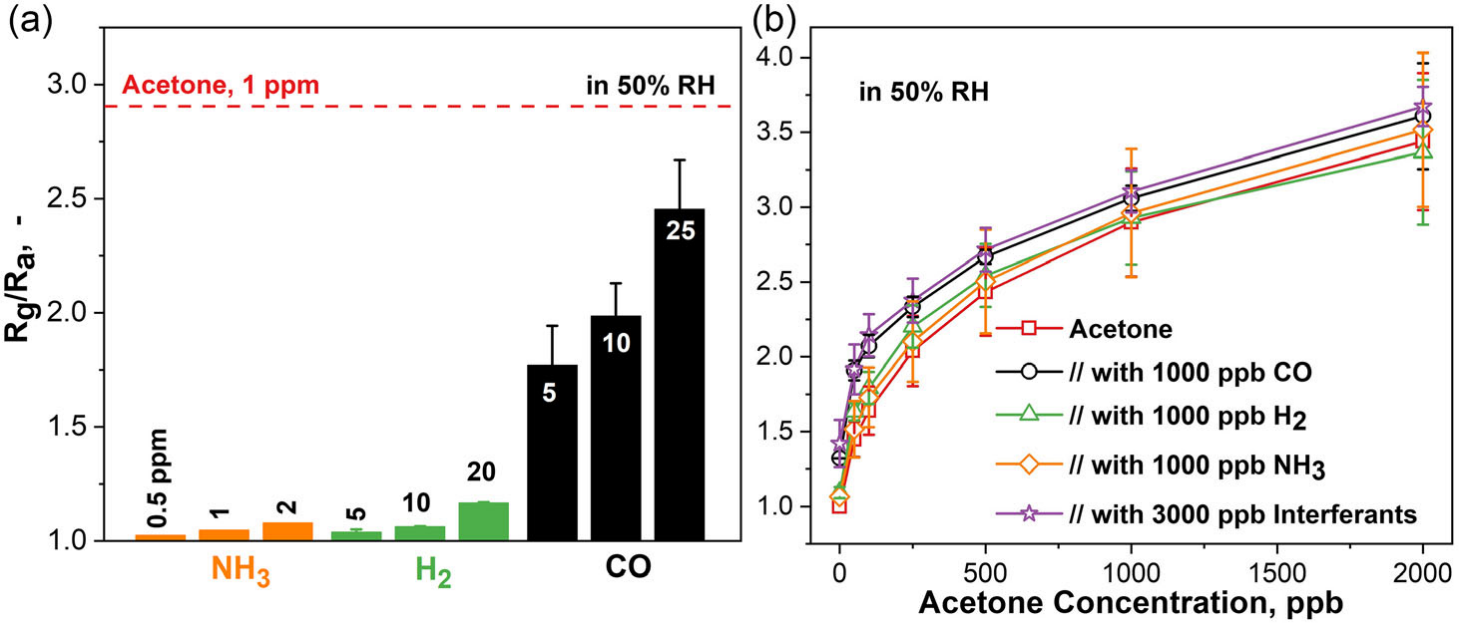
a) Responses of the CeO_2_–CuO sensor upon exposure to 0.5, 1, and 2 ppm NH_3_ (orange) and 5, 10, and 20 (25) ppm of H_2_ (green) and CO (black). The average response to 1 ppm acetone is indicated (red dashed line) for reference. b) Corresponding responses to 50–1500 ppb acetone without (squares) and with inorganic interferants (CO (circles), H_2_ (triangles) NH_3_ (diamonds), and a mixture (stars)). Note that 50% RH instead of 90% RH was used due to a limitation of the measurement setup. The error bars indicate the standard deviations for *N* = 3 identically prepared sensors.

Besides acetone, exhaled breath might contain also highly concentrated inorganic interferants (e.g., up to 1.8 ppm NH_3_,^[^ ^34^ ^]^ 20 ppm H_2_^[^ ^35^ ^]^ and 25 ppm CO^[^ ^36^ ^]^). Therefore, the sensor performance when exposed to 0.5–2 ppm NH_3_, 5–20 ppm H_2_, and 5–25 ppm CO was investigated (**Figure** **2**a). Most impressively, the sensor's response to 1000 ppb acetone (red dashed line) was consistently higher even than that of orders of magnitude higher concentrated 2000 ppb NH_3_, 20 000 ppb H_2_, and 25 000 ppb CO. This was found consistently for three identically prepared sensors (error bars, Figure 2a), depicting good reproducibility of ±10%. Most importantly, the sensor featured high selectivity of 30 toward NH_3_, 100 toward H_2_, and 9.6 toward CO, being the highest to our knowledge for such low‐temperature sensors, and the only ones reported in humid conditions (Table 1).

Importantly, the CeO_2_ clusters not only enhanced the humidity robustness, but at the same time contributed to an overall improvement in acetone selectivity over that of pure CuO (Figure S4a,b, Supporting Information, e.g., also showing the selectivity to isoprene and ethanol). Note that all sensor measurements were performed at 150 °C, being the optimal compromise between high sensor response, selectivity, and response and recovery times (*τ*_res_ and *τ*_rec_, Figure S4a and S5a,b, Supporting Information). Specifically, a decrease in acetone sensor response (i.e., 46% and 57%) and selectivity (i.e., 2 and 1 (isoprene), 3.15 and 2.9 (CO), 37 and 73 (H_2_)) was observed for 125 and 200 °C, respectively. At the same time, *τ*_res_ and *τ*_rec_, were significantly increased at lower temperatures (*τ*_res_ from 670 to 1200 s). In addition, it is worth noting that CeO_2_–CuO sensor can also be used for the detection of ethanol (Figure S4a, Supporting Information). In cases where ethanol detection is undesired (e.g., for acetone detection in breath analysis), it can however be removed using an upstream room‐temperature catalytic 3 mol% Pt‐Al_2_O_3_ filter^[^ ^37^ ^]^ (see Supporting Information on material preparation and filter assembly). This filter combusts interfering ethanol to sensor‐inert species, while maintaining most of the acetone (i.e., 15% reduction). The high reactivity at room temperature is attributed to the presence of well‐dispersed Pt clusters,^[^ ^37^ ^]^ while the selective combustion of interferants over acetone may come from the Al_2_O_3_ support^[^ ^38^ ^]^ that converts alcohols preferentially over acetone.^[^ ^39^ ^]^ This way, the acetone selectivity toward ethanol could be improved by >95% (Figure S6, Supporting Information). This sensor detects also CH_4_ and H_2_S (Figure S7, Supporting Information, normalized response of 0.1 and 0.14 at 1 ppm, respectively) but features much smaller signals compared to acetone (i.e., 90% and 86%, respectively). Note that despite the good selectivity, CuO sensors are known to deteriorate upon prolonged exposure to H_2_S (i.e., forming CuS^[^ ^40^ ^]^), so this sensor may not be suitable for use in halitosis, where H_2_S can reach up to 0.5 ppm.^[^ ^41^ ^]^

To challenge the sensor even more, and as human breath is a complex mixture of various analytes, its performance was evaluated in gas mixtures. Figure 2b shows the response to 50–2000 ppb acetone without (squares) and with inorganic interferants (NH_3_ (diamonds), H_2_ (triangles), CO (circles), and their mixture (stars)). Most importantly, the addition of interfering molecules hardly changed the acetone response for three identically prepared sensors. As expected, based on sensor selectivity (Figure 2a), the highest deviation in sensor response was observed upon addition of 1000 ppb CO (i.e., 12%), followed by H_2_ and NH_3_ (<10%) for 500 ppb acetone. At a higher concentration of 1000 ppb, acetone deviations decreased further (<10%) for all binary interferant mixtures. The mixture containing 3000 ppb of inorganic interferants showed a similar deviation (i.e., 14%) as observed for CO.

Overall, the minimal deviations in sensor performance in the presence of complex inorganic mixtures are remarkable. This further promotes the sensor's suitability as a low‐power alternative in real environments and highlights the merit of doped p‐type sensors.

### 2.2 Effect of Material Properties on the Sensing Behavior

**Figure 3:**
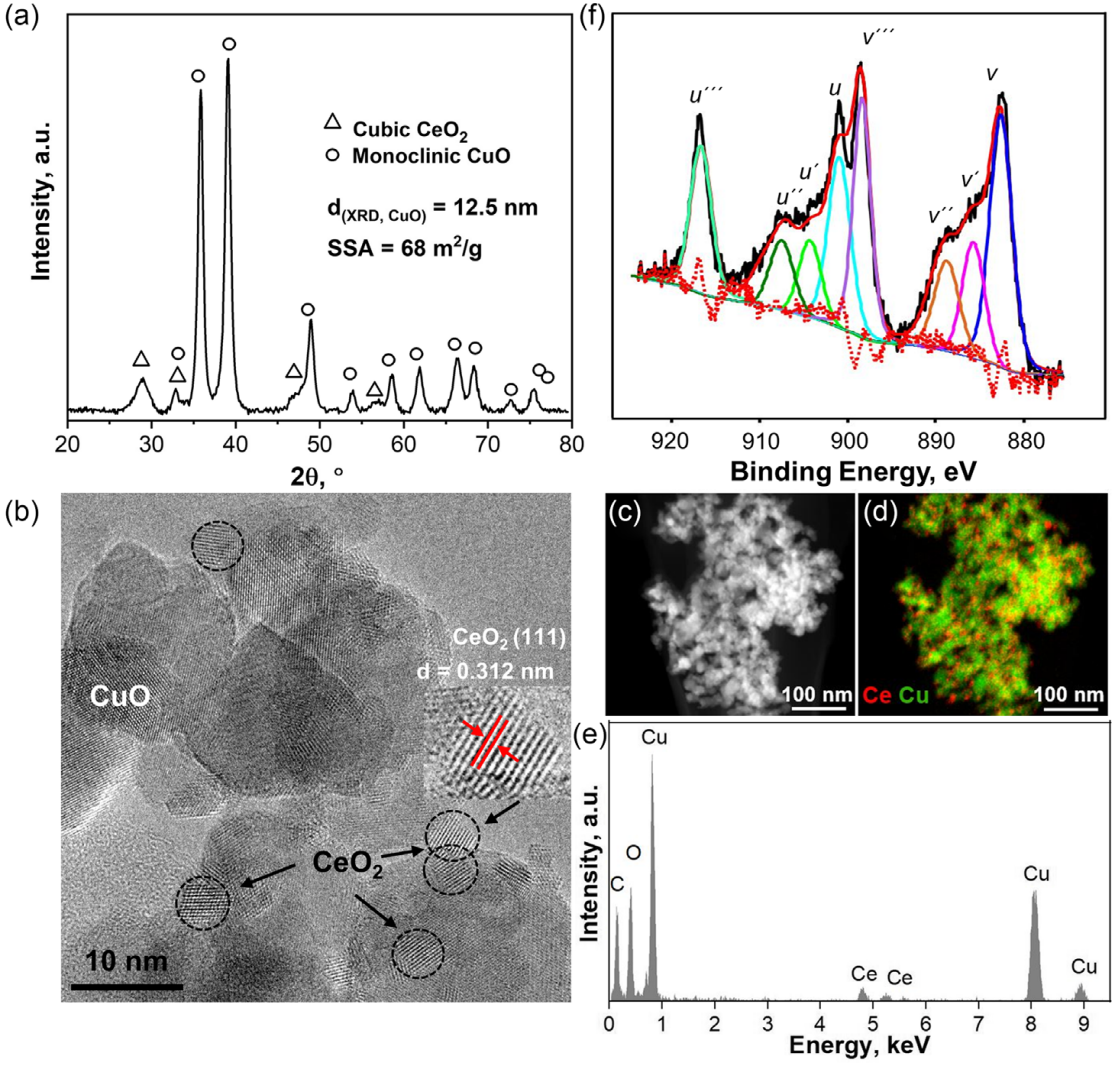
a) XRD pattern of the CeO_2_–CuO powder with reference peaks for monoclinic CuO (circles) and cubic CeO_2_ (squares). The CuO crystal size (*d*_XRD_) and the SSA are indicated. b) HRTEM image of the nanoparticles, with the inset showing the lattice fringes corresponding to the CeO_2_ (111) crystal plane. c,d) HAADF‐STEM images of CeO_2_–CuO nanoparticles with e) EDXS analysis of the Ce (red) and Cu (green) atoms. f) The deconvoluted XPS spectra of the Ce 3d profile indicate the Ce^3+^ and Ce^4+^ oxidation states present in the nanoparticles.

To better understand the sensor performance,^[^ ^26^ ^]^ the CeO_2_–CuO nanomaterial characteristics were investigated by XRD, N_2_ adsorption, and TEM, while the chemical composition was studied by XPS (**Figure** **3**). The XRD pattern shows distinct peaks indicating the presence of cubic CeO_2_ (Figure 3a, triangles, reference peak positions indicated by symbols) and monoclinic CuO (circles) with an average crystal size (*d*_XRD_) of 3.9 and 12.5 nm, respectively, according to Rietveld refinement. This indicates that Ce^4+^ is the predominant oxidation state, while suboxides may be present, as shown in the XPS data. Note that due to both low signal‐to‐noise ratios and broad peaks of the CeO_2_ crystal structure in the XRD spectra, the particle size distribution of CeO_2_ nanoparticles was determined additionally with high‐resolution transmission electron microscopy (HRTEM). The CuO crystal size extracted from the CeO_2_–CuO pattern is in agreement with literature for flame‐made CuO (i.e., 10^[^ ^42^ ^]^ and 11 nm^[^ ^43^ ^]^), although under slightly different conditions (i.e., as‐prepared CuO compared to annealed CeO_2_–CuO, where CeO_2_ inhibits grain growth). Note that the XRD pattern together with crystal size (i.e., 13.6 nm) for pure annealed CuO is shown in Figure S8, Supporting Information. The average particle size (*d*_BET_) of the annealed CeO_2_–CuO determined by N_2_ adsorption assuming spherical particles is 13.5 nm, suggesting predominantly monocrystallinity, in agreement with literature after particle annealing.^[^ ^44^ ^]^ This small crystal size (hence high surface area) is beneficial for enhanced sensor sensitivity, as shown with SnO_2_ sensors.^[^ ^45^ ^]^

Figure 3b shows an HRTEM image of the CeO_2_–CuO nanoparticles. Spherical particles decorated with smaller clusters are visible. The presence of lattice fringes indicates high crystallinity. The lattice spacing of the smaller clusters (i.e., 0.312 nm) matches well the CeO_2_ (111) plane (Figure 3b, inset). Hence, the smaller clusters correspond to CeO_2_ (*d*_TEM_ of 2 to 5 nm). Meanwhile, the larger particles correspond to CuO (distinguishable also by the lattice spacings of the (−111, 111) planes) with *d*_TEM_ ranging from 10 to 12 nm, respectively, in agreement with the crystal size (*d*_XRD_) of CuO extracted from the CeO_2_–CuO XRD pattern. The size distribution of CeO_2_ nanoparticles is shown in Figure S9, Supporting Information, with a geometric diameter (*d*_g_) and standard deviation (*σ*_g_) of 3.2 nm and 0.805, respectively. These results are supported by high‐angle annular dark‐field scanning transmission electron microscopy (HAADF‐STEM) imaging (Figure 3c,d), where smaller CeO_2_ (or ceria suboxide) clusters are dispersed on the CuO nanoparticles in agreement with the corresponding energy‐dispersive X‐ray spectroscopy (EDXS) mapping (Figure 3e).

As the oxidation state of ceria has been shown to influence the humidity robustness of ceria‐based sensors,^[^ ^21^ ^]^ we performed XPS analysis with our nanomaterials. Figure 3f shows the XPS spectra of the Ce 3d profile with eight peaks, where v's and u's represent the Ce 3d_5/2_ and Ce 3d_3/2_ contributions, which in turn reveal the presence of both Ce^3+^ (denoted as v′ and u′ at 884 and 902 eV, respectively) and Ce^4+^.^[^ ^46^ ^]^ Note that by assuming no enrichment of Ce^4+^ over Ce^3+^ or vice versa, the area of the u″′ peak in the total Ce 3d region (d_3/2_ and d_5/2_) can be used to describe the relative amount of Ce^4+^ in the material, in this case only 11%, and is in good agreement with literature on humidity‐robust CeO_2_–In_2_O_3_ sensors (9.97%^[^ ^21^ ^]^). In fact, altering the Ce content (i.e., to 1 and 10 mol% Ce) leads to a loss in humidity robustness, as well as increased response times, as shown in Figure S10, Supporting Information (XRD spectra in Figure S8, Supporting Information). The sensitivity to OH species comes from the water vapor interactions with the ionosorbed oxygen on the sensor surface, which results in the formation of terminal hydroxyl groups and a surface site for chemisorption of oxygen (S). This in turn leads to an increase in sensor resistance,^[^ ^29^ ^]^ as is visible in Figure S10b, Supporting Information
(1)

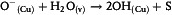




In the presence of Ce^4+^ metal ions, a reduction to Ce^3+^ and hydrogen ions (H^+^) is possible^[^ ^21^ ^]^

(2) $$4Ce^{4+}+2H_{2}O_{\left(v\right)}\to 4Ce^{3+}+4H^{+}+O_{2}$$

This redox process is beneficial for the regeneration of the CuO surface, where the formed Ce^3+^ and H^+^ scavenge hydroxyl groups formed between the CuO and the water vapor

(3) $$OH_{\left(Cu\right)}+Ce^{3+}+H^{+}\to Ce^{4+}+H_{2}O_{\left(v\right)}$$

Hence, high humidity robustness is achieved at high Ce^4+^ metal ion concentrations, as was observed for the 5 mol% CeO_2_–CuO sensor (Figure 1a and S10a, Supporting Information). To further tune the Ce^4+^/Ce^3+^^[^ ^21^ ^]^ ratio one could employ FSP.^[^ ^47^ ^]^ Further information regarding the chemical composition (i.e., oxygen [from 525 to 535 eV] and copper [from 930 to 968 eV] species) is shown in Figure S11, Supporting Information. Species related to the lattice (*O*_a_: 529.6 eV), nonlattice (*O*_b_: 531.5 eV, i.e., the defect oxide or surface oxygen ions in low coordination situations and weakly bound oxygen species), and adsorbed (*O*_c_ at 533 eV, i.e., hydroxyl species) oxygen,^[^ ^48^ ^]^ together with the paramagnetic state of Cu^2+^ (i.e., CuO: shake‐up features at 945 and 965 eV),^[^ ^49^ ^]^ were present in the CeO_2_–CuO nanoparticles. Note that although different oxygen species^[^ ^50^ ^]^ are known to contribute to the sensing performance, the presence of specifically oxygen vacancies (*O*_b_, i.e., acetone detection with Ce–In_2_O_3_^[^ ^21^ ^]^), as well as the Ce^4+^/Ce^3+^ ratio, have proven beneficial in the regeneration of the sensing surface, both features present in the ceria–copper oxide nanoparticles.

### 2.3 Breath Analysis

**Figure 4:**
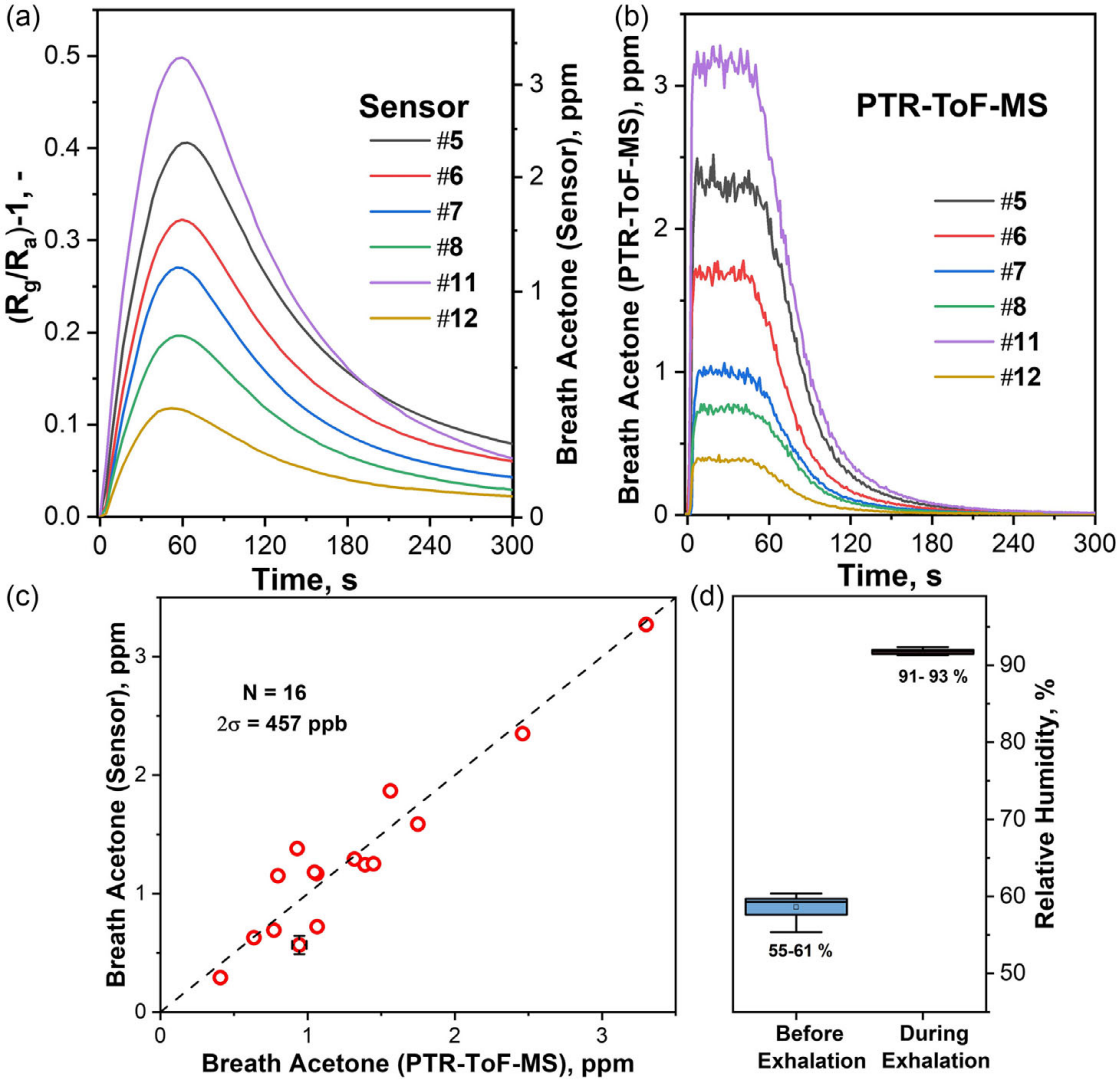
a) Sensor response (left ordinate) and respective acetone concentrations (right ordinate) when exposed to a breath pulse of volunteers #5, #6, #7, #8, #11, and #12. b) Acetone concentrations for each respective volunteer as detected by the PTR‐ToF‐MS. c) Scatter plot indicating the breath acetone measured by the sensor and the PTR‐ToF‐MS of all 16 volunteers (*N* = 16). The ideal sensor–MS correlation (dashed line) and precision (2*σ*, according to Bland‐Altman) are indicated. The error bars indicate the deviation for the single data points of the acetone concentrations detected by the sensor and the PTR‐ToF‐MS, between the four breath samples analyzed from one volunteer. d) Box plot depicting the change in relative humidity as detected by the SHT2x humidity sensor before and during exhalations of all 16 volunteers.

As a proof of concept, the sensor's performance was evaluated on real human breath of 16 overnight‐fasted (>9 h) volunteers. Breath sampling was standardized for all volunteers using a tailor‐made breath sampler that allows for reproducible breath sampling and buffering of end‐tidal air for ≈60 s.^[^ ^51^ ^]^ Each volunteer provided one breath sample, which was simultaneously analyzed by the CeO_2_–CuO sensor and bench‐top PTR‐ToF‐MS. **Figure** **4**a shows the sensor response for six volunteers after a single breath exhalation. The sensor exhibits distinctly different response maxima, which are reflected by the PTR‐ToF‐MS data (Figure 4b). In fact, applying the nonlinear power–law calibration to the sensor (see Figure 4a, right ordinate, as determined on the flow‐bench) allows us to compare the acetone concentrations detected by the sensor with the PTR‐ToF‐MS, being, for example, 3280 ppb on the sensor and 3300 ppb on the PTR‐ToF‐MS for volunteer #11. Noteworthy are the different dynamics of the sensor measurement compared to the PTR‐ToF‐MS. While the PTR‐ToF‐MS shows an acetone plateau that lasts for 60 s (corresponding to the buffering of breath inside the sampling tube), the sensor reaches a maximum response after 60 s. This should be attributed to the longer sensor response time (340 s) compared to the buffering (60 s).

The acetone concentrations of all volunteers detected by both instruments are shown in Figure 4c and ranged from 300 to 3150 ppb, which is in agreement with previous studies on 30 healthy volunteers (i.e., from 148 to 2744 ppb, though without overnight fasting^[^ ^12^ ^]^). Most importantly, the sensor was in good agreement with PTR‐ToF‐MS (dashed line in Figure 4c), having a Pearson's coefficient of *r* = 0.95 and a bias (accuracy) and precision (2*σ*) of 90 and 457 ppb, respectively, according to Bland–Altman analysis (see Figure S12, Supporting Information).^[^ ^52^ ^]^ This is comparable to or better than studies that quantified acetone concentrations with a sensor, specifically an adsorption column (2*σ* = 3237 ppb^[^ ^53^ ^]^), an enzymatic sensor (2*σ* = 3052 ppb^[^ ^54^ ^]^), a chemoresistive Si/WO_3_ sensor (442 ppb^[^ ^55^ ^]^), and even a sensor array consisting of a Pt/WO_3_ and SnO_2_ sensor (645 ppb^[^ ^56^ ^]^). Note, however, that the current measurements were conducted with a single sensor at low temperature.

Note that this remarkable bias and precision was achieved despite the presence of other breath volatiles, for example, isoprene (73–536 ppb, Table S2, Supporting Information) and ethanol (270–400 ppb). While in our controlled setting, the ethanol varied only slightly, it could reach higher concentrations in some cases (e.g., during hand disinfection), affecting the sensor precision. In this case, a catalytic packed bed Pt/Al_2_O_3_ filter^[^ ^37^ ^]^ can be used to mitigate the ethanol problem (as shown in Figure S6, Supporting Information). It is worth noting that the sensor was also robust to abrupt changes in RH before and during the exhalations, as is illustrated in Figure 4d.

Owing to this sensor's good precision, it is suitable to resolve fine acetone differences, as is necessary for most applications such as dieting (like ketogenic diets, 700–2500 ppb^[^ ^8^ ^]^), and treatment of metabolic diseases (like epilepsy, 700–2500 ppb^[^ ^10^ ^]^). Moreover, one of the volunteers gave four subsequent breath samples, and a rather small deviation of 77 ppb (i.e., ±10%) was observed with the sensor (error bar in Figure 4c), showing good reproducibility. Note that acetone concentrations according to PTR‐ToF‐MS also changed by 48 ppb (i.e., ±5%) over the period of these measurements. This observation further supports the potential viability of this sensor as a suitable low‐power alternative to operate in breath applications. In the future, such low‐power sensors may be used even as a part of self‐powered healthcare devices,^[^ ^57^ ^]^ as has been reported on the example of capacitive acetone sensors.^[^ ^58^ ^]^

## 3 Conclusion

A low‐power and low‐cost flame‐made copper oxide sensor containing cerium oxide (CeO_2_–CuO) was presented for the selective detection of acetone and ethanol. The unique advantages of this p*‐*type sensor are its low operation temperature (150 °C), high sensitivity (i.e., down to 50 ppb), humidity robustness (i.e., <10% fluctuation in response between 10 and 90% RH), as well as high selectivity toward inorganic interferants. This allowed us to measure acetone in the presence of orders of magnitude higher inorganic NH_3_, H_2_, and CO gases, as might be present in exhaled human breath. The high sensitivity was attributed to the high surface area and typically highly porous structure of such directly deposited flame‐made sensors, comprising small (i.e., *d*_XRD_ = 12.5 nm) CuO nanoparticles containing CeO_2_ clusters. The partial humidity robustness of the sensor was attributed to the presence of the CeO_2_ clusters, particularly the high fraction of Ce^4+^ (that can be reduced to Ce^3+^ at high humidity values), as determined with XPS analysis, which may act as OH^−^ scavenger and oxygen generator.^[^ ^21^ ^]^ Most importantly, under standardized conditions, this sensor showed excellent performance even when tested on the exhaled breath of 16 volunteers. In fact, it was capable of detecting the entire range (i.e., from 300 to 3150 ppb) with a good bias and precision of 90 and 457 ppb. Thus, by addressing the sensitivity and humidity cross‐sensitivity challenges of p‐type sensors (e.g., CeO_2_–CuO), they can become a viable low‐temperature alternative for device integration (e.g., battery‐driven devices) in various application including breath acetone monitoring in mobile healthcare applications.

## 4 Experimental Section

4.1

4.1.1

##### Nanoparticle and Sensor Film Preparation

Composite ceria–copper oxide nanoparticles^[^ ^59^ ^]^ were prepared by FSP with a precursor solution containing cerium(II)‐ethyl hexanoate (Alfa Aesar, 12 wt% Ce)^[^ ^60^ ^]^ and Soligen Copper 8 (OMG Borchers GmbH, 8.04 wt% Cu) dissolved in a 1:2 v/v mixture of xylene (AnalaR NORMAPUR, ≥98.5%) and 2‐ethylhexanoic acid (Sigma Aldrich, >99%). The total final metal content (i.e., 5 mol% Ce and 95 mol% Cu) was 0.25 M. The 5 mol% Ce loading was chosen based on previous literature with CeO_2_–In_2_O_3_ sensors^[^ ^21^ ^]^ that showed humidity robust sensing performance at similar Ce loadings. The precursor solution was supplied at 5 mL min^−1^ through the FSP nozzle and dispersed (1.5 bar pressure drop) with 5 L min^−1^ oxygen to a fine spray ignited and sustained by premixed methane/oxygen (1.25/3.2 L min^−1^) with additional sheath oxygen (5 L min^−1^). The ceria–copper oxide nanoparticles were directly deposited^[^ ^25^ ^]^ onto Al_2_O_3_ sensor substrates (15 × 13 × 0.8 mm, Electronic Design Center, Case Western Reserve University) featuring interdigitated electrodes (spacing 350 μm) and a Pt heater on the backside.^[^ ^61^ ^]^ The sensor substrate was placed at 20 cm height above the burner (HAB) during sensing particle deposition and shortly after it was lowered to 14.5 cm HAB for 30 s in situ annealing by FSP with pure xylene^[^ ^62^ ^]^ to improve the mechanical stability of the just‐deposited sensing film. For further thermal stabilization, the sensing films were air annealed in an oven (Carbolite Gero GmbH, 30–3000 °C) at 500 °C for 5 h.^[^ ^62^ ^]^

For characterization with XRD, N_2_ adsorption, XPS, and TEM, nanoparticles were collected on a water‐cooled glass microfiber filter (Albert‐Hahnemuehle GF‐6, 25.7 cm in diameter) downstream of the sensing substrates with a vacuum pump (Seco SV 1025C, Busch) at 50 cm HAB. These nanoparticles were removed from the filter with a spatula, sieved (250 μm mesh), and thermally stabilized by annealing in air at 500 °C for 5 h in an oven (Carbolite Gero GmbH, 30–3000 °C). Similarly, 0, 1 and 10 mol% Ce‐loaded FSP‐prepared CuO nanoparticles^[^ ^42^ ^]^ and directly deposited sensor films were prepared as reference materials.

##### Material Characterization

A Bruker AXS D8 Advance diffractometer, operated at 40 kV and 30 mA at 2*θ* (Cu K_α_) = 10 to 80° at a scanning step size and speed of 0.1° and 0.097° s^−1^, respectively, was used for analysis by XRD. Crystal phases were identified with reference structural parameters of monoclinic CuO (Tenorite, PDF 005‐0661) and cubic CeO_2_ (Fluorite, PDF 004‐0593). The corresponding crystal sizes (*d*_XRD_) were determined by Rietveld refinement analysis using the Topas 4.2 (Bruker) software. The specific surface area (SSA) of the nanoparticles was determined by N_2_ adsorption (Brunauer–Emmett–Teller, Micromeritics Tristar 3000). Prior to analysis, samples were degassed for 1 h at 200 °C under N_2_. The corresponding diameter assuming spherical particles (*d*_BET_ = 6/(SSA × *ρ*_CuO_)) was calculated using the density of Tenorite CuO (*ρ*_CuO_ = 6.52 g cm^−1^).

For the XPS investigation, the data was acquired on a PHI Quantera II spectrometer. The samples were analyzed using a microfocused monochromatic Al X‐ray source (50.6 W) over an area of ≈200 μm. Data was recorded at pass energies of 280 eV for survey scans, and 55 eV for the high‐resolution scan with 1 and 0.1 eV step sizes, respectively. Charge neutralization of the sample was achieved using a combination of both low‐energy electrons and argon ions. The Au 4f electron at 84.0 eV was used as a standard reference to calibrate the photoelectron energy shift. Spectra in Ce 3d (region from 870 to 930 eV), Cu 2p (region from 925 to 970 eV), and O 1s (region from 525 to 535 eV) were collected and fit to identify the ratio of Ce and Cu in different oxidation states and the types of oxygen species present in the sample. The data analysis was performed with the PHI MultiPak software (version: 2.8C, 2007‐9‐04).

For electron microscopy imaging, particles were dispersed in ethanol (EtOH) and deposited onto molybdenum (Mo) grids. The HRTEM images were acquired on a JEM‐ARM300F (GrandARM, JEOL) operated at 300 keV. Furthermore, a HAADF‐STEM (Talos F200X, Thermo Scientific) operated at 200 kV and equipped with an EDXS detector was used to image the CeO_2_–CuO particles. The area‐derived particle diameters were determined from HRTEM images using ImageJ (version 1.53c) to measure the ceria–copper oxide particle/cluster areas assuming spherical particles. To differentiate between the two particle compositions, the d‐spacing calculated from the visible lattice fringes corresponding to the CeO_2_ (111) and CuO (−111, 111) planes were used.

##### Sensor Characterization

Two sensors were tested simultaneously in a chamber^[^ ^61^ ^]^ and gas‐mixing system described previously,^[^ ^63^ ^]^ equipped with four mass flow controllers (MFCs, Bronkhorst) for analyte gases. The sensors were heated to 150 °C (unless specified otherwise) by supplying the appropriate DC voltage through the substrate heater.

Dry synthetic air (C_*n*_H_m_ and NO_*x*_ ≤ 100 ppb, Pan Gas) was used as a carrier gas. Acetone (15 ppm), hydrogen (H_2_, 50 ppm), carbon monoxide (CO, 500 ppm), ammonia (NH_3_, 10 ppm), isoprene (500 ppm), H_2_S (10 ppm), CH_4_ (10 ppm), and EtOH (15 ppm, all Pan Gas, in synthetic air) were admixed by calibrated MFCs to obtain the gas mixtures at a total flow rate of 300 mL min^−1^. To achieve the desired RH, synthetic air was bubbled through a 125 mL glass vessel (Drechsel bottle, sintered glass frit, Sigma‐Aldrich) filled with ultrapure water (Milli‐Q A10, Merck) and validated with a humidity sensor (Sensirion, SHT2*x*, SHT3*x* & SHTC1/W1). Heated (55 °C) inert Teflon tubing was used to connect the MFCs with the sensor chamber to avoid condensation. Measurements were carried out at 90% RH, unless otherwise specified. The sensor response, *S*, to each analyte was calculated as

(4) $$S=\frac{R_{\text{analyte}}}{R_{\text{air}}}$$

where $R_{\text{analyte}}$ and $R_{\text{air}}$ represent the film resistances during exposure in air with and without the analyte, respectively.^[^ ^28^ ^]^ The sensor response (*τ*_res_) and recovery (*τ*_rec_) times were the times needed to reach or recover 90% of the resistance change during or after analyte exposure, respectively. The acetone selectivity was defined as the ratio between the acetone response and that to a specific analyte following IUPAC guidelines,^[^ ^64^ ^]^ being (*R*_acetone_−1)/(*R*_interferant_−1). The signal‐to‐noise ratio (SNR) was defined as the ratio of the analyte signal to the noise measured in synthetic air.

##### Human Breath Sampling and Analysis

A group of 16 volunteers (6 female and 10 male), aged 22–33, participated in this study. All volunteers were nonsmoking, healthy, and free from known cardiovascular, respiratory, or metabolic diseases (for detailed information, see Table S1, Supporting Information). All volunteers were instructed to abstain from alcohol and vigorous exercise 24 h before the breath test and not to use chemical mouthwash less than 2 h before the test. Measurements were carried out after overnight fasting (>9 h). Each volunteer was informed about the protocol and gave written consent. This study was not subject to an ethics approval, as confirmed by the responsible authority (ETH Zürich Ethikkommission).

Breath samples were collected with a tailor‐made sampler.^[^ ^51^ ^]^ Specifically, each volunteer exhaled within 5 s through a sterile and disposable mouthpiece (EnviteC‐Wismar GmbH, Germany) into the inert and heated (60 °C) sampler (tube volume: 270 mL). The end‐tidal breath was fed to the sensor through an inert Teflon transfer line at a flow rate of 125 mL min^−1^ using a micropump (Schwarzer Precision, Germany). The moisture content of each breath sample was measured with a humidity sensor (SHT2x, Sensirion AG). In addition, a PTR‐ToF‐MS 1000 (Ionicon, Austria) was used to validate the sensor results simultaneously. The PTR‐ToF‐MS drift voltage, temperature, and pressure at measurement were 600 V, 60 °C, and 2.3 mbar, respectively. The H_3_O^+^ ions served as primary ions and analyte concentrations were measured in counts per second at mass‐to‐charge (*m*/*z*) ratios of 59.05 (acetone),^[^ ^65^ ^]^ 69.07 (isoprene),^[^ ^66^ ^]^ and 47.05 (ethanol),^[^ ^65^ ^]^ respectively. Before each set of measurements, the PTR‐ToF‐MS was three‐point calibrated with the above gas standards over the relevant range.

##### Statistical Analysis

No preprocessing of the data was done. The sample size (*N*) for each statistical analysis was indicated. Experimental measurements (data) repeated under the same conditions (including, at least, three replicas) were presented as mean ± standard deviation (*σ*). The standard deviation was calculated for three identically produced and tested sensors to determine the precision of the sensor response. Statistical Bland‐Altman analysis^[^ ^52^ ^]^ (Figure S12, Supporting Information) was conducted to assess the sensor's agreement with PTR‐ToF‐MS. The average difference between the sensor and MS was defined as the bias, and the precision corresponded to two times the standard deviation (2*σ*). The software OriginPro 2018 G (OriginLab Corporation, USA) was used for the statistical analysis.

## Conflict of Interest

The authors declare no conflict of interest.

## Supporting information

Supplementary Material

## Data Availability

The data that support the findings of this study are available from the corresponding author upon reasonable request.
