# Supplementary material for: A Strategy to Enhance Humidity Robustness of p‐Type CuO Sensors for Breath Acetone Quantification
Source: Small Sci. 2023 Feb 28;3(4):2200096. doi: 10.1002/smsc.202200096 (PMC11935944; doi:10.1002/smsc.202200096)
Supplement: Supplementary file 1 — Supplementary Material [file SMSC-3-2200096-s001.pdf]

Supporting Information

A strategy to enhance humidity robustness of *p*-type CuO sensors for breath acetone quantification

Dina N. Oosthuizen[a], *Ines C. Weber* [a,b]*

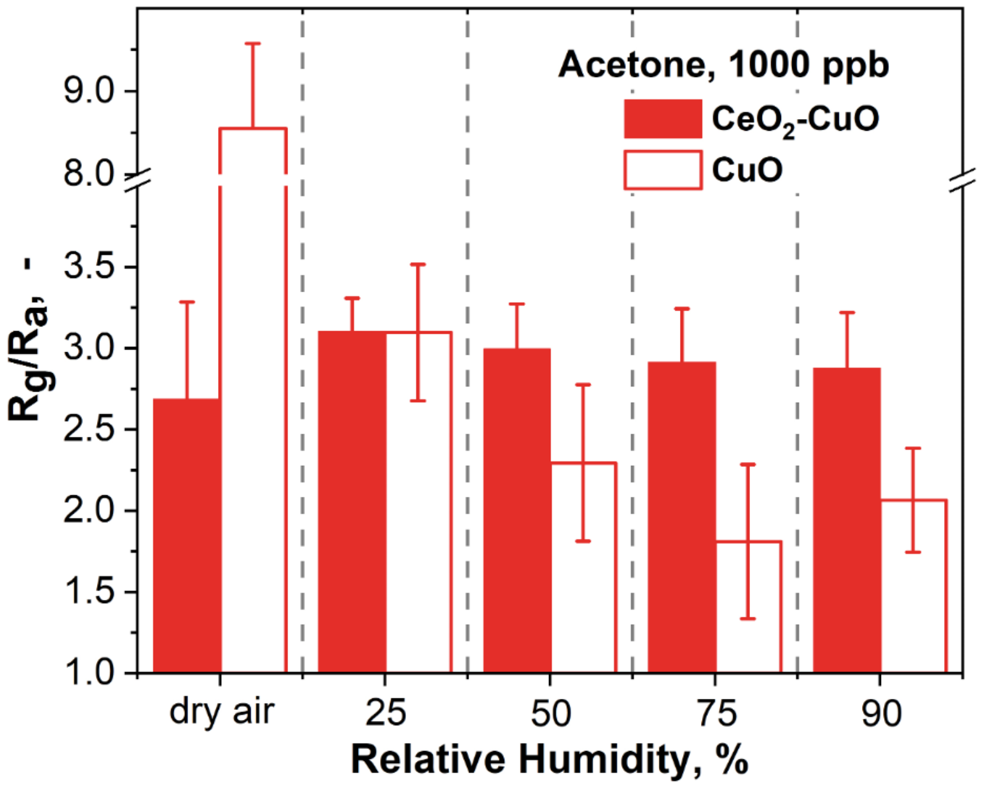

Figure S1: Sensor responses towards 1000 ppb acetone as a function of RH with the CeO_2-CuO (closed bars) and CuO sensors (open bars). Error bars represent N = 3 identically prepared sensors.

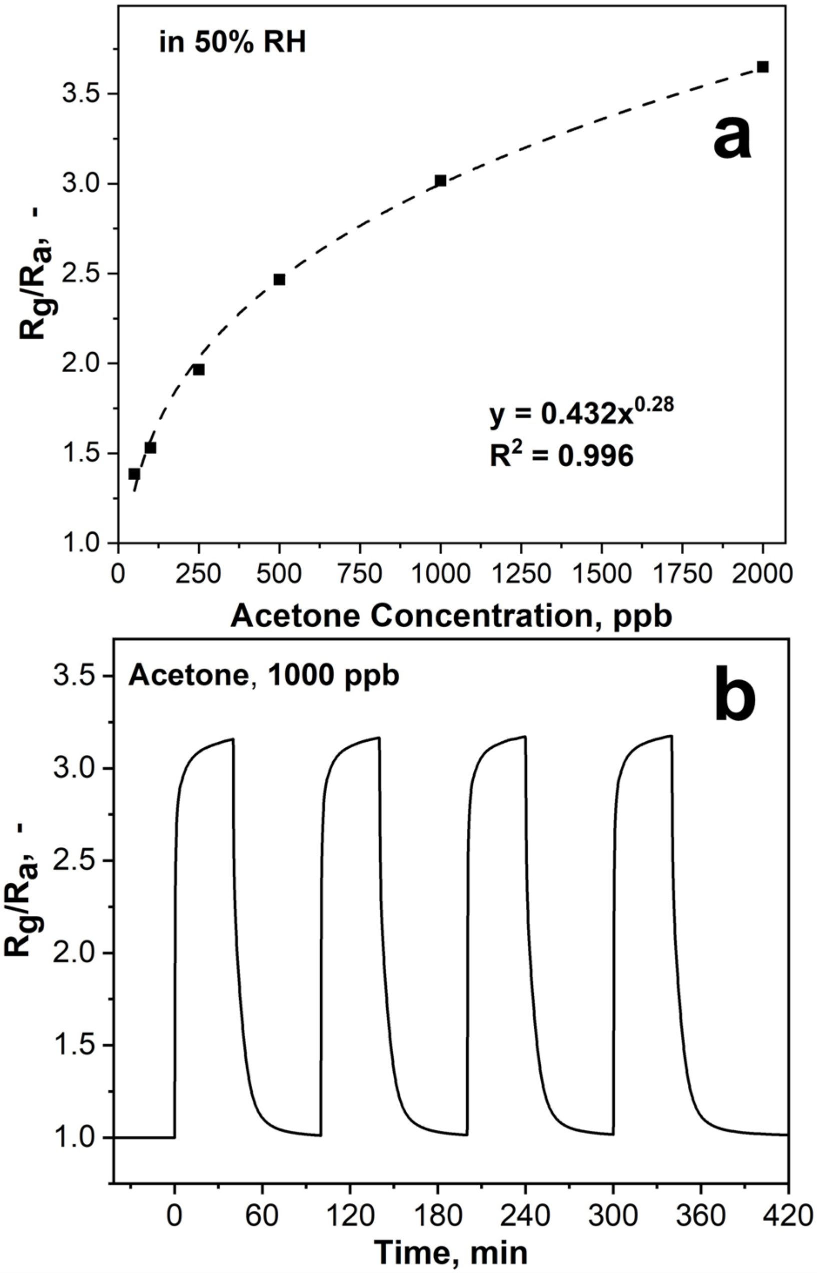

Figure S2: (a) Non-linear dependence of sensor response to acetone concentrations (50–2000 ppb) in 50% RH. **(b)** Response to four consecutive pulses of 1000 ppb acetone in 90% RH.

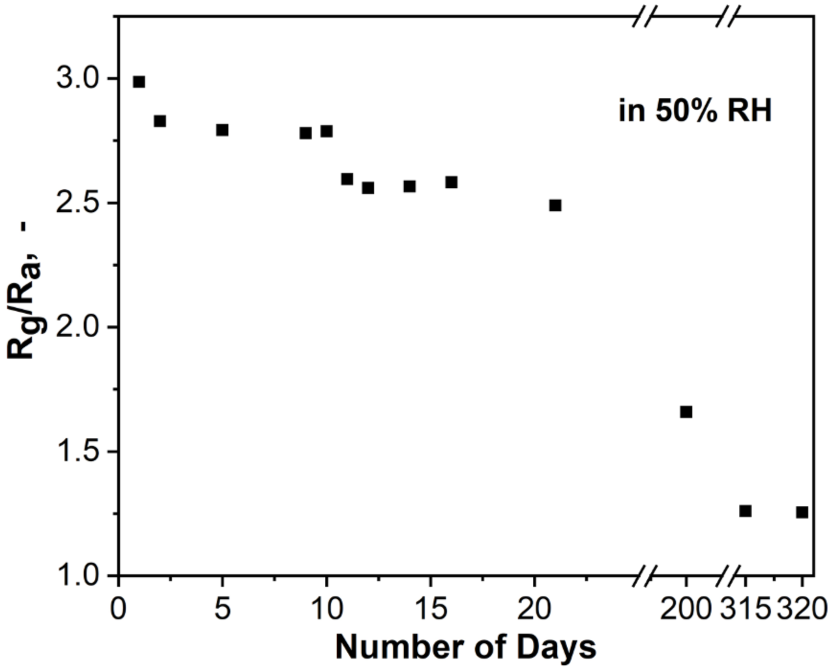

Figure S3: Sensor response to 1 ppm acetone over time in 50% RH.

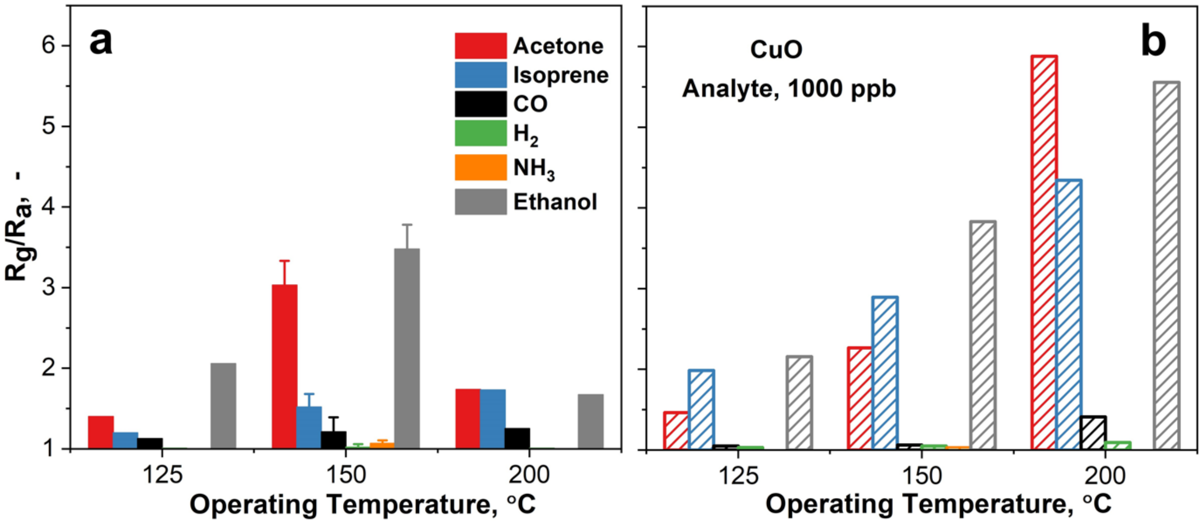

Figure S4: (a) CeO_2-CuO and **(b)** CuO sensor responses towards 1000 ppb acetone (red), isoprene (blue), CO (black), H_2 (green), NH_3 (orange), and ethanol (grey) in 90% RH at three operating temperatures. Error bars represent N = 3 identically prepared sensors.

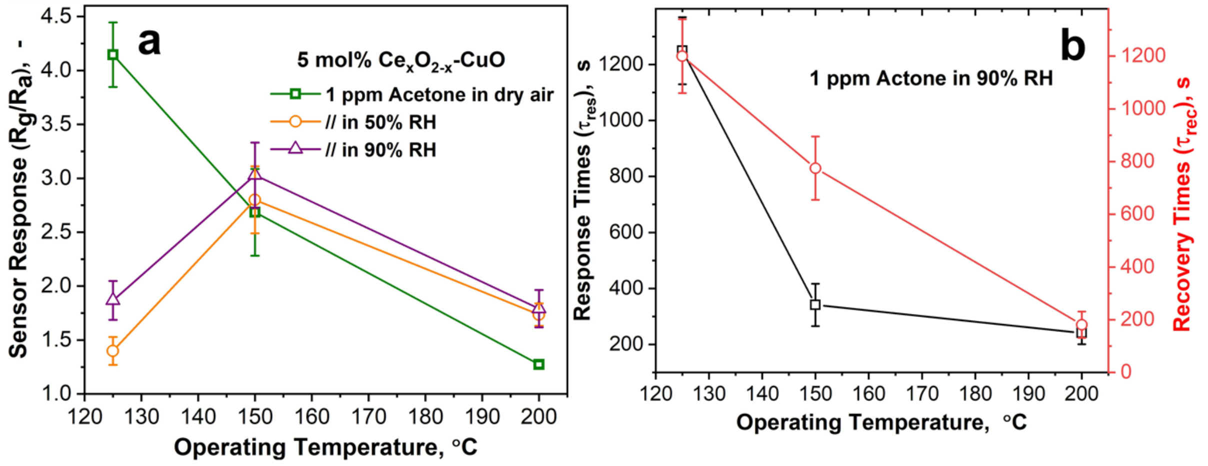

Figure S5: (a) CeO_2-CuO sensor response towards 1000 ppb acetone at 125, 150, and 200 °C in dry air (squares), 50% RH (circles), and 90% RH (triangles). **(b)** Response (squares) and recovery times (circles) to 1000 ppb acetone at different operating temperatures for three identically produced sensors at 90% RH. Error bars represent N = 3 identically prepared sensors.

Pt/Al_2O_3 filter fabrication

The 3 mol% Pt/Al_2O_3 [68] nanoparticles for packed bed filters were prepared by FSP. The packed bed catalytic filter was prepared by packing 30 mg of 3 mol% Pt/Al_2O_3 nanoparticles inside a compact Teflon tube (i.e. 4 mm inner tube diameter, 1.5 cm packed bed length) and closing both sides with wool and quartz sand.[86]

Pt/Al_2O_3 filter-sensor testing

The packed bed filter was connected downstream of the gas mixing unit and upstream of the sensors. For comparison to previous work,[69] an optimal total flow rate of 150 mL min^{-1} was selected for the measurements. The exhaust gas from the packed bed was analyzed with a PTR-ToF-MS 1000 (Ionicon, Austria). The sensor-filter setup was exposed to each analyte gas for 30 minutes, before it was subjected again to synthetic air.

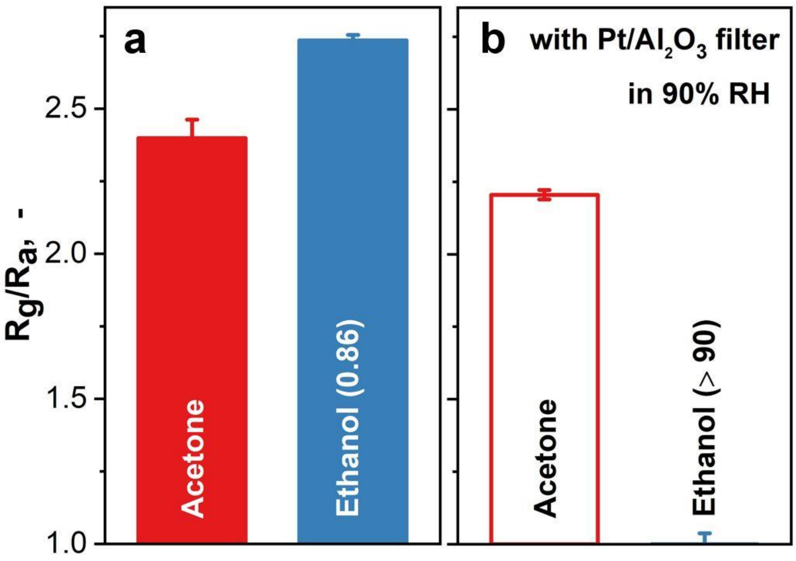

Figure S6: CeO_2-CuO sensor responses **(a)** without (closed bars) and **(b)** with (open bars) a catalytic Pt/Al_2O_3 packed bed filter (at room temperature) towards 1000 ppb acetone (red) and ethanol (blue) in 90% RH. The acetone selectivity is indicated in brackets. Error bars indicate the standard deviations of N = 3 subsequent measurements.

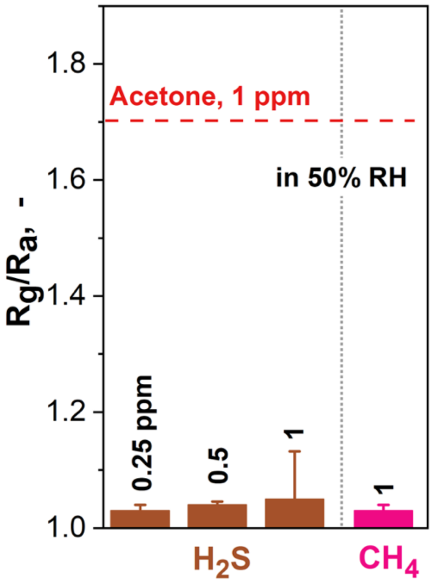

Figure S7: Normalized responses of the CeO_2-CuO sensor upon exposure to 0.25, 0.5 and 1 ppm H_2S (brown) and 1 ppm of CH_4 (pink) in comparison to 1 ppm acetone (normalized, dashed line) in 50% RH.

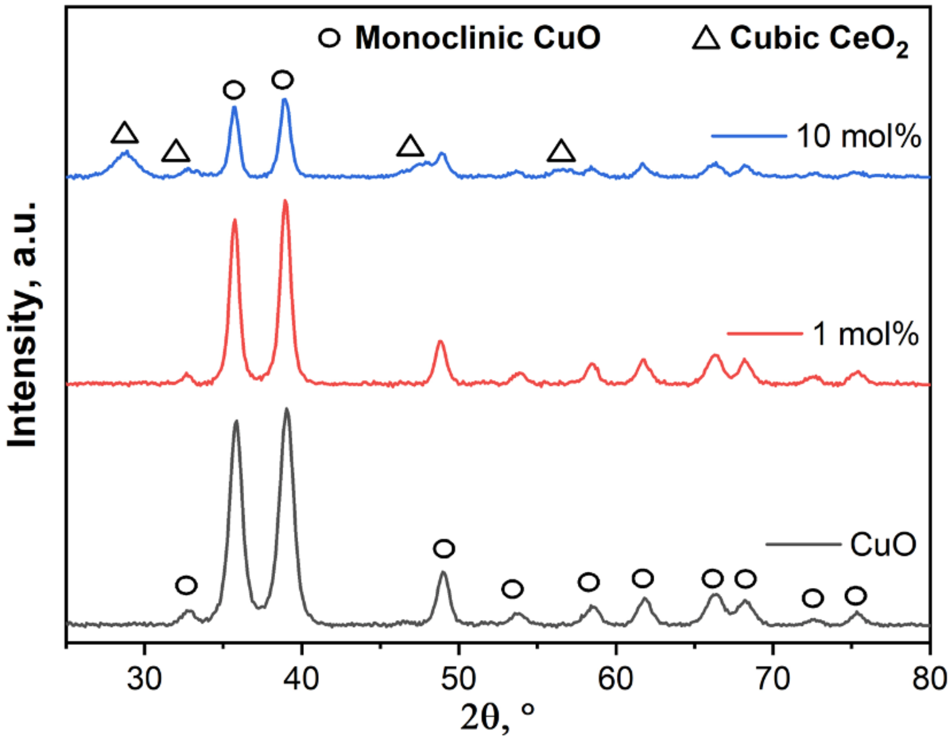

Figure S8: XRD pattern of the pure CuO powder (black), as well as 1 mol% (red) and 10 mol% (blue) Ce-CeO_2, with reference peaks for monoclinic CuO (circles) and cubic CeO_2 indicated. The crystal sizes (d_{XRD}) are indicated.

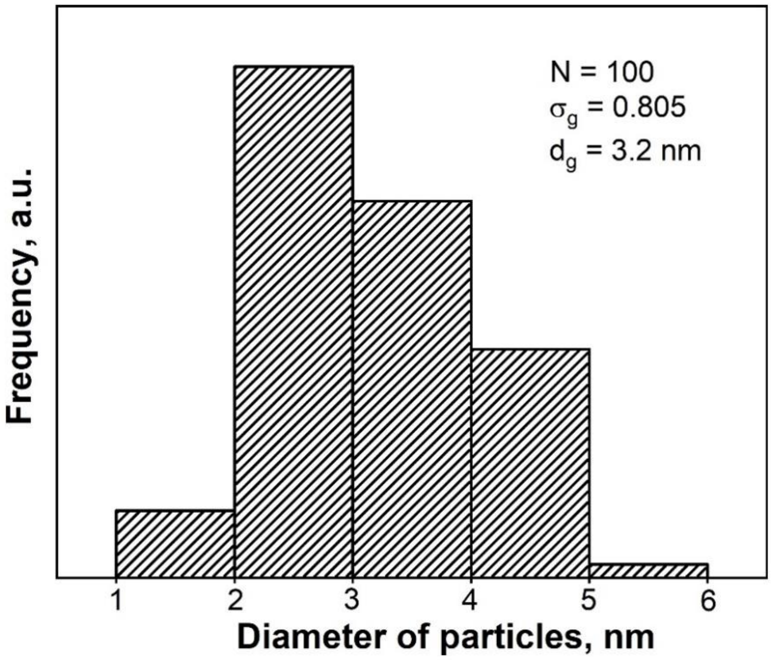

Figure S9: Size distribution of CeO_2 clusters as determined from several HRTEM images, with the mean geometric diameter (d_g), standard deviation (σ_g) and number (N) of counted particles.

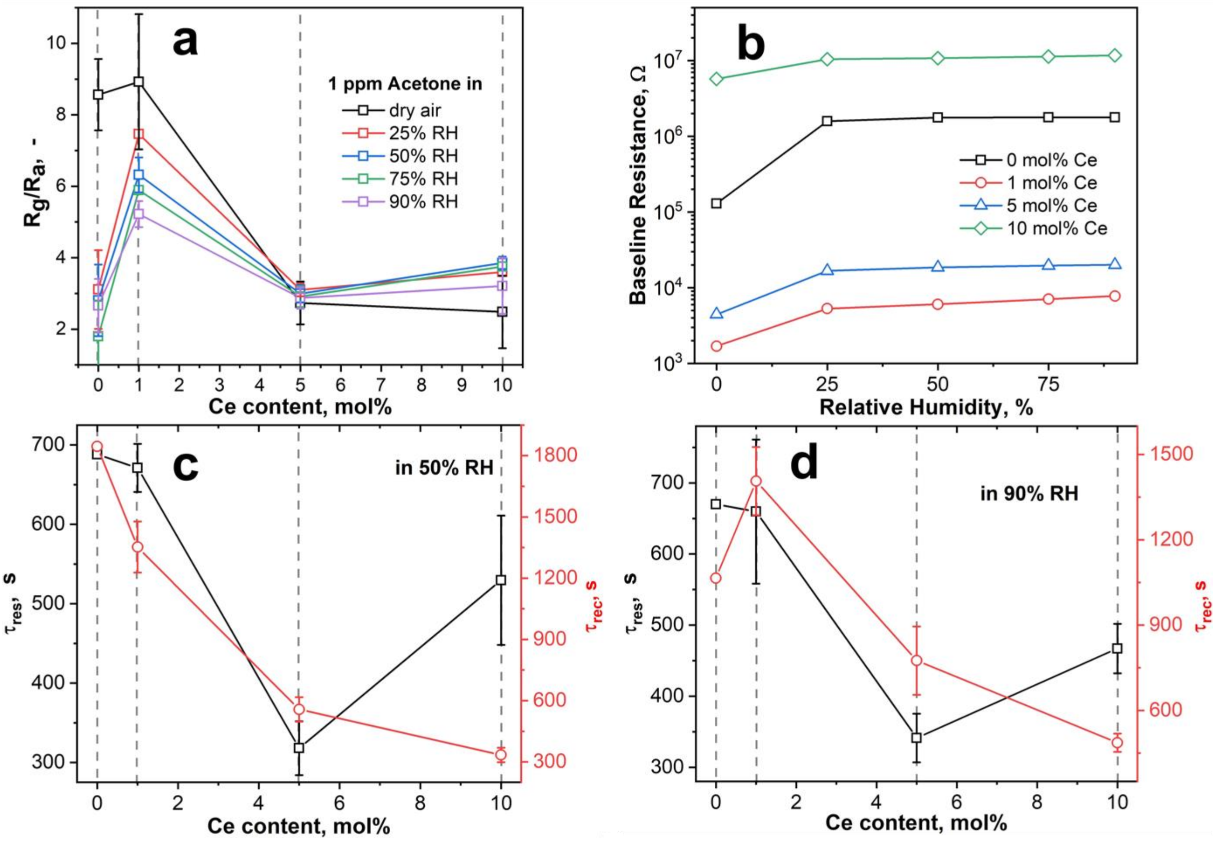

Figure S10: (a) Sensor response when exposed to 1 ppm acetone for 0, 1, 5 and 10 mol% Ce-CuO in dry air (black), as well as 25 (red), 50 (blue), 75 (green) and 90% (purple) RH. **(b)** Sensor resistance in air at 0, 25, 50, 75 and 90% RH for pure CuO (squares), as well as 1 (circles), 5 (triangles) and 10 (diamonds) mol% Ce loadings. **(c)** Sensor response (squares, left ordinate) and recovery times (circles, right ordinate) at different Ce loadings in 50% RH and **(d)** 90% RH. Error bars indicate the standard deviations of N = 3 subsequent measurements for 1 and 10 mol% Ce-CuO and N = 3 identically prepared sensors (i.e., CuO and 10 mol% Ce-CuO).

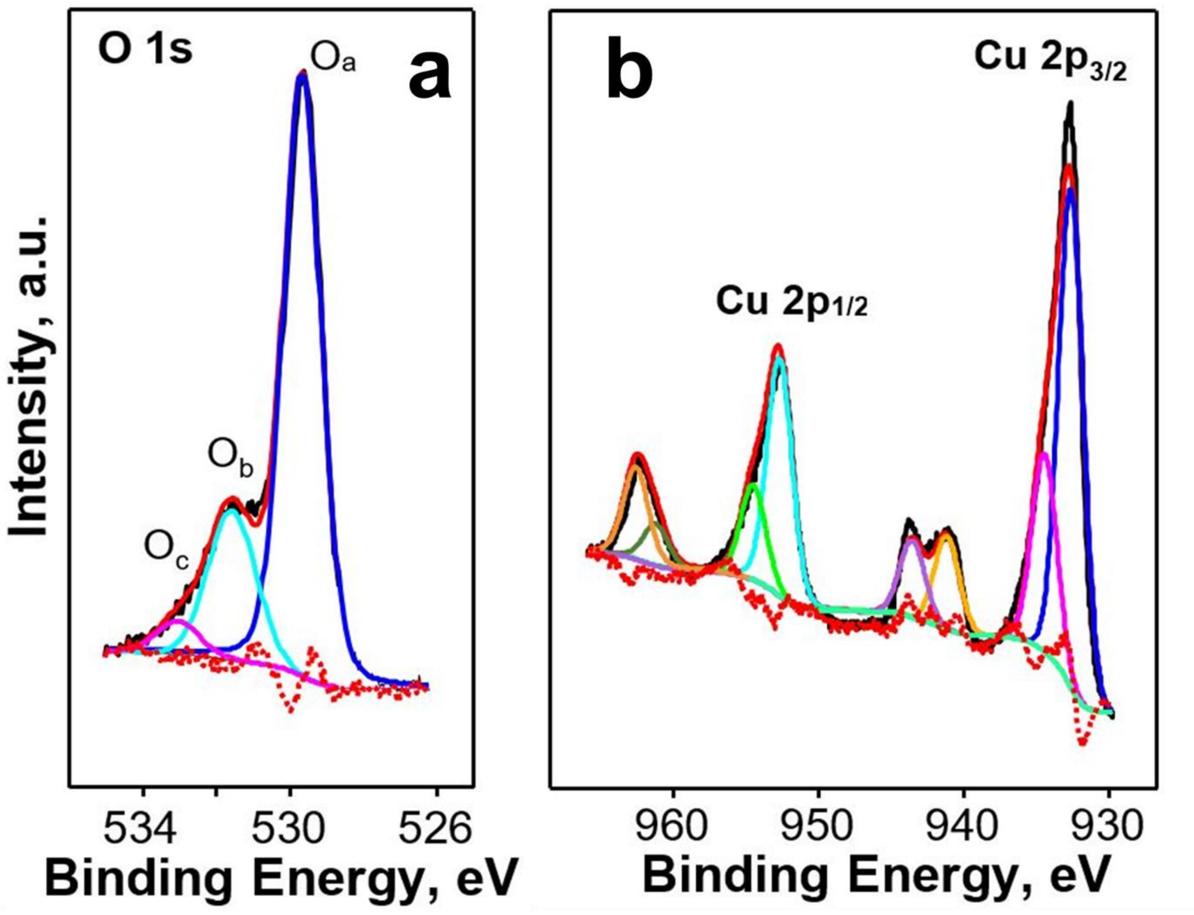

Figure S11: XPS spectra of the **(a)** O 1s and **(b)** Cu 2p profiles, indicating the different oxygen species and copper cations present in the CeO_2-CuO nanoparticles.

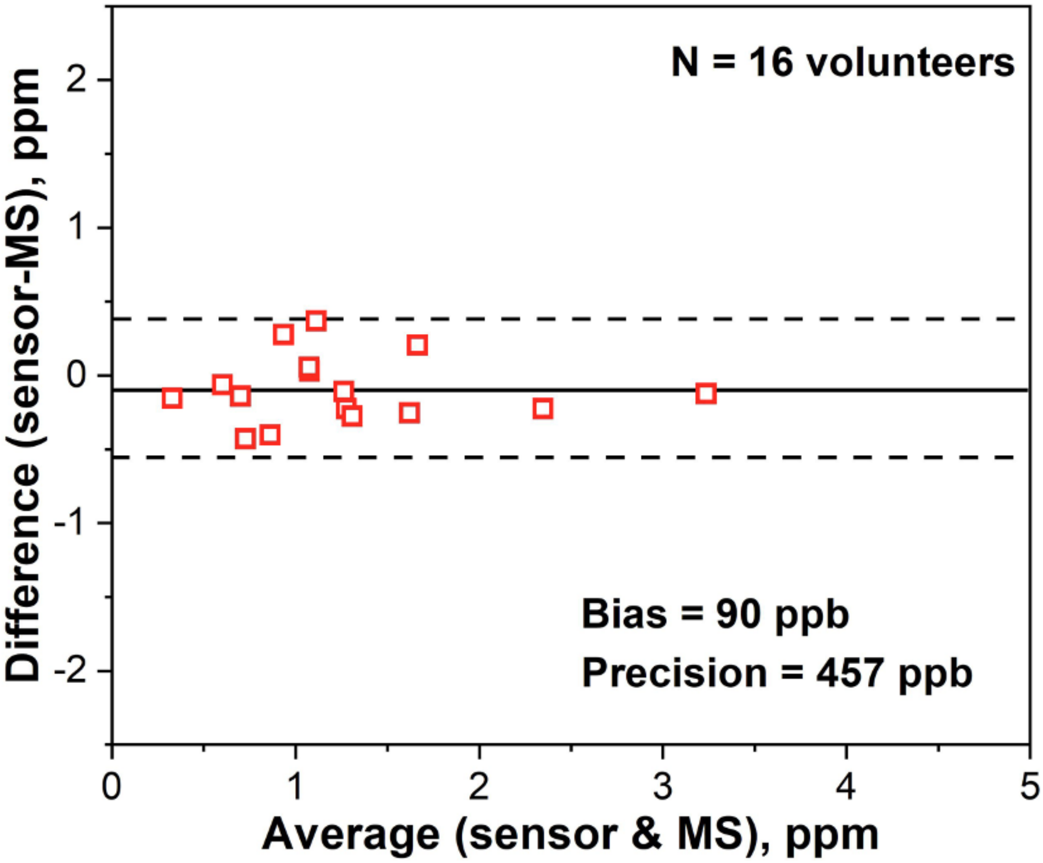

Figure S12: Bland-Altman analysis between breath acetone concentrations by the PTR-ToF-MS and the sensor as a function of N = 16 samples (16 volunteers).

Table S1: Data of the volunteers for the breath analysis study

ID number	Gender	Age	Height (cm)	Weight (kg)	Smoker (y/n)
1	M	31	175	64	n
2	F	28	167	57	n
3	F	25	162	55	n
4	M	24	185	85	Occasionally (4 days prior)
5	F	28	169	55	n
6	M	25	175	78	n
7	M	26	180	70	n
8	F	33	175	63	n
9	F	22	180	81	n
10	M	30	192	90	n
11	M	23	166	64	n
12	M	25	190	90	n
13	M	23	193	79	n
14	M	28	184	78	n
15	M	24	184	76	n
16	F	33	166	80	n

Table S2: Breath ethanol and acetone concentrations of each volunteer as measured by PTR-ToF-MS.

ID number	PTR-ToF-MS		
	**Acetone (ppm)**	**Ethanol (ppm)**	**Isoprene (ppm)**
1	0.80	0.27	0.27
2	1.56	0.29	0.09
3	0.93	0.30	0.26
4	1.06	0.37	0.27
5	2.46	0.29	0.14
6	1.75	0.30	0.36
7	1.05	0.31	0.29
8	0.77	0.37	0.43
9	0.64	0.27	0.16
10	1.39	0.40	0.09
11	3.30	0.33	0.54
12	0.41	0.29	0.41
13	1.45	0.31	0.38
14	1.32	0.39	0.23
15	1.06	0.33	0.13
16	0.94	0.34	0.07
